# Four‐and‐one‐half years' experience in monitoring of reproducibility of an MR spectroscopy system — application of *in vitro* results to interpretation of *in vivo* data

**DOI:** 10.1120/jacmp.v15i3.4754

**Published:** 2014-05-08

**Authors:** Agnieszka Skorupa, Magdalena Wicher, Tomasz Banasik, Ewa Jamroz, Justyna Paprocka, Aleksandra Kiettyka, Maria Sokót, Marek Konopka

**Affiliations:** ^1^ Department of Medical Physics Maria Sklodowska‐Curie Memorial Cancer Center and Institute of Oncology Gliwice Poland; ^2^ Scanx Medical Imaging Sp. z o.o. Katowice Poland; ^3^ Henryk Niewodniczański Institute of Nuclear Physics Polish Academy of Sciences Kraków Poland; ^4^ Helimed Diagnostic Imaging Sp. z o.o. Katowice Poland; ^5^ Child Neurology Department Silesian Medical University Katowice Poland

**Keywords:** magnetic resonance spectroscopy, reproducibility, Canavan disease

## Abstract

The primary purpose of this work was to assess long‐term *in vitro* reproducibility of metabolite levels measured using ^1^H MRS (proton magnetic resonance spectroscopy). The secondary purpose was to use the *in vitro* results for interpretation of ‘H MRS *in vivo* spectra acquired from patients diagnosed with Canavan disease. ^1^H MRS measurements were performed in the period from April 2006 to September 2010. 118 short and 116 long echo spectra were acquired from a stable phantom during this period. Change‐point analysis of the *in vitro* N‐acetylaspartate levels was exploited in the computation of *f_T_* factor (ratio of the actual to the reference N‐acetylaspartate level normalized by the reciprocity principle). This coefficient was utilized in the interpretation of *in vivo* spectra analyzed using absolute reference technique. The monitored time period was divided into six time intervals based on short echo *in vitro* data (seven time intervals based on long echo *in vitro* data) characterized by *f_T_* coefficient ranging from 0.97 to 1.09 (based on short echo data) and from 1.0 to 1.11 (based on long echo data). Application of this coefficient to interpretation of *in vivo* spectra confirmed increased N‐acetylaspartate level in Canavan disease. Long‐term monitoring of an MRS system reproducibility, allowing for absolute referencing of metabolite levels, facilitates interpretation of metabolic changes in white matter disorders.

PACS numbers: 87.19.lf, 87.61.Tg, 87.64.K‐, 87.64.kj

## I. INTRODUCTION

H1 MRS *in vivo* has many important applications in the study of human brain biochemistry. In recent years our group utilized this technique in the evaluation of metabolic changes in various neurological disorders in children (including neurometabolic ones).^(1)^ As neurometabolic disorders individually are extremely rare, it was necessary to pool the data acquired during a relatively long period of time. Thus, long‐term reproducibility of the technique was of crucial importance to provide information about its sensitivity. As it has already been shown, reproducibility depends on the quantification method used.^(2)^

H1 MRS *in vivo* is often claimed to be a quantitative technique; however, absolute metabolite level quantification is not straightforward. In the normal brain, water content and relaxation properties of the water molecules are known — thus, the water signal can be used as an internal standard in the calculations of metabolite concentrations.^(3)^ In such an approach, several sources of error (such as B1 inhomogeneity, changes in coil loading conditions, changes in hardware performance) are eliminated; however, in the case of the diseased brain, accurate referencing to brain water is quite complex. Because water relaxation times have been shown to change in many neurometabolic disorders, it could be misleading to scale the metabolite levels accordingly to not fully relaxed water signal, as some researchers have done in application to long TE spectra from tumors^(4)^ or from white matter hyperintensities in the elderly.^(5)^ However, T2 measurements are required to be elaborate enough to account for compartmentation of water into several pools of different spin‐spin relaxation times (in the normal brain: cerebrospinal fluid, extra/intracellular water, myelin water).^(6)^ Interestingly, the number of these pools may increase in the pathologic brain.^(7)^ Apart from changes in water relaxation properties, the water content is subject to change in many neurometabolic disorders. Although the signal corresponding to the respective water compartments could be referenced to the signal from an external standard located next to the patient's head, this method is valid only under the assumption of negligible B1 changes between these two measurement locations.^(8)^

In the absolute standard quantification technique, the *in vivo* metabolite levels are referenced to the *in vitro* signals acquired during an independent measurements session from a phantom with a known content located centrally in the coil. However, B1 inhomogeneity, time‐dependent hardware changes, and variations in coil loading conditions should be taken into consideration.® The importance of phantom measurements has already been recognized in H1 MRS quality control procedures devoted to establishment of reproducibility of the technique.^9^, ^10^, ^11^, ^12^, ^13^ Although the results of these measurements cannot be directly translated to *in vivo* conditions, it is usually more practical to assess reproducibility under ideal conditions. Many sources of variability inherent to *in vivo* measurements are eliminated using phantoms, yet time dependent changes in the scanner performance are easier to detect in *in vitro* conditions. Moreover, the results from *in vitro* experiments can be used to correct *in vivo* data for the detected instability.^(12)^

The primary aim of this work was to assess long‐term *in vitro* reproducibility of metabolite levels obtained using H1 MRS. Traditionally, results of reproducibility monitoring of MRS systems are expressed using coefficients of variations^10^, ^11^ or are presented using traditional control charts.^(12)^ We propose application of a change‐point analysis^(14)^ to detect break points when significant changes in the performance of MRS system occur. Since the phantom measurements were performed as a part of quality assurance program in H1 MRS *in vivo* studies of neurological disorders, the secondary aim of this work was to utilize the *in vitro* results in the retrospective interpretation of *in vivo* data.

## II. MATERIALS AND METHODS

## A. H1 MRS

H1 MRS measurements were performed over the time period from April 2006 to September 2010 on a 1.5T GE scanner (GE Healthcare, Waukesha, WI) equipped with a transmit/receive head coil.

### A.1 H1 *MRS in the studies of neurological disorders in children*

The studied children were admitted for etiologic diagnostic evaluation (or diagnosis verification) because of the suspicion of neurometabolic disorder due to clinical features or abnormal laboratory results. Based on detailed metabolic and neuroimaging screening, several patients were diagnosed with neurometabolic disorders.^(1)^ In this work we present the results obtained from two children diagnosed with Canavan disease (case 1 aged 8 months and case 2 aged 18 months) and 46 children (median age: 24 months, age range: 2‐47 months) diagnosed with other neurological disorders (epilepsy, developmental delay, and cerebral palsy).

Both water suppressed and water unsuppressed spectra were recorded. The acquisition parameters were as follows: TE 35 and 144 ms, TR 1500 ms, volume of interest: 8 ml, bandwidth 2500 Hz, samples 2048, number of signal averages: 128 (for water suppressed spectra) and 16 (for water unsuppressed ones), localization sequence: PRESS. We intended to measure six spectra for each TE from the volumes of interest located bilaterally in the frontal, periventricular (laterally to the ventricles) and in parieto‐occipital areas. Due to motion of the patients and time constraints, the number of brain localizations examined was reduced in some patients. This number ranged from one to six for each TE. The spectra measured from all localizations were pooled in the analysis.

LCModel software analyzes H1 MRS *in vivo* spectra as linear combinations of individual metabolites *in vitro* spectra (basis set).^(15)^ The basis set used in this work was measured at another site and was provided with the software. We focused on the N‐acetylaspartate (NAA) level determined separately for short and long TE using absolute standard calibration. In this method, the measured *in vivo* spectra are multiplied by $f_{\text{scale}}$ factor:

(1) $$f_{scale}=f_{tra}\cdot f_{gain}\cdot f_{calib}\cdot f_{t}\cdot \frac{1}{V}$$

The coefficient $f_{tra}=10^{0.005\cdot (TG-65)}$ (where TG denotes transmitter gain) is related to the principle of reciprocity (higher coil loading requires higher TG for 90° excitation which results in a lower signal intensity received from the same number of protons) and is used for coil loading correction.^16^, ^17^ TG was set automatically during preset procedures.

The coefficient $f_{gain}=2^{\left(6-\frac{R1}{2})+(30-R2\right)}$ (where R1 and R2 denote digital and analogue receiver gains, respectively) is used for receiver gain correction.

The coefficient $f_{calib}=\frac{c_{true}}{c_{lcm}}$ (where $c_{\text{true}}$ denotes true metabolite concentration in the phantom, while $c_{\text{lcm}}$ denotes the metabolite level output by LCModel (adjusted for the voxel size, TG ‐ using $f_{tra}$, R1 and R2 ‐ using $f_{gain}$)) is used to correct for scaling differences between the basis set and *in vivo* data. According to the LCModel manual, NAA was dissolved in the standard solvent (1 mM TSP, 72 mM $K_{2}{\text{HPO}}_{4}$, 28 mM ${\text{KH}}_{2}{\text{PO}}_{4}$, 200 mM sodium formate, 1g/L ${\text{NaN}}_{3}$) and a reference spectrum was measured from the phantom located centrally in the coil.^(18)^ This measurement was performed on 16 November 2007.

The coefficient $f_{T}$ accounts for changes in the temporal setup of the scanner. This coefficient may change suddenly (for example, after breakdown of the system, after replacement of hardware parts, after parameters readjustment) or gradually (for example, as hardware parts wear down). Determination of $f_{T}$ factor is described in Material and Methods section A.2 below.

*V* denotes voxel size.

No correction for T1 metabolite relaxation and for differences between *in vivo* and *in vitro* relaxation times was performed in the absolute reference technique.

Since water signal intensity is susceptible to pathological white matter changes, the NAA levels obtained with a water scaling technique are presented to show the shortcomings of using nonfully relaxed water signal as a reference in neurometabolic disorders. In this technique the measured spectra are multiplied by $f_{\text{scale}}$ factor according to the equation:^(18)^

(2) $$f_{scale}=\frac{Area_{met}}{N1HMET\cdot Conc_{met}}\cdot \frac{2\cdot Conc_{water}\cdot ATT_{water}}{Area_{water}}$$

where ${\mathrm{Area}}_{\mathrm{met}}$ and ${\mathrm{Conc}}_{\mathrm{met}}$. are the observed resonance areas and concentrations of the selected singlet in the basis set (by default, corresponding to the methyl (${\text{CH}}_{3}$) groups of creatine); *N1HMET* is the number of equivalent protons contributing to this singlet; and ${\mathrm{Area}}_{\mathrm{water}}$, ${\mathrm{Conc}}_{\mathrm{water}}$, and ${\mathrm{ATT}}_{\mathrm{water}}$ are the observed resonance areas, concentration (35.8 M) and attenuation factor of water signal due to transverse relaxation with T2=80 ms. Of note, the assumed values for ${\text{Conc}}_{\text{water}}$ and ${\text{ATT}}_{\text{water}}$ relate to normal adult brain, and accurate values were not determined individually for each patient. No correction for T1 metabolite and water relaxation and for differences between *in vivo* and *in vitro* metabolite relaxation times was performed in water scaling technique.

The results obtained for Canavan disease were compared to those obtained in other neurological disorders by means of z‐scores computed according to the formula:

(3) $$z=\frac{m-m_{mean}}{m_{\sigma }}$$

where *m* denotes the NAA level observed in Canavan disease, $m_{\mathrm{mean}}$ denotes the mean NAA level obtained in other neurological disorders, and $m_{\sigma }$ denotes standard deviation of the NAA level in other neurological disorders. To minimize the influence of age‐dependent NAA changes, the z‐scores for case 1 were calculated using the mean and standard deviation computed for children aged from 2 to 12 months, while the z‐scores for case 2 were obtained using the mean and standard deviation computed for children aged from 12 to 24 months. Differences characterized by the z‐scores lower than ‐2.0 SD or higher than 2.0 SD were considered to be statistically significant. Age‐matching was mandatory because age‐related variation is an important source of the total variance in MRS studies in young children.^19^, ^20^

Direct comparison of NAA levels between CD and other neurological disorders is motivated by uniqueness of NAA changes observed in CD. While most pathological conditions (such as ischemia, brain injury, brain cancer or multiple sclerosis) are characterized by decreased NAA or NAA‐to‐Cr ratio, CD involves accumulation of NAA in the brain.^(21)^ Although comparison of the NAA levels in CD to those observed in healthy children would provide a better insight into the pathophysiology of CD, spectroscopic datasets acquired from healthy, age‐matched children are difficult to obtain due to ethical reasons.

### A.2 *Determination of the coefficient* $f_{T}$

A standard spherical brain phantom provided by the manufacturer was used for long‐term reproducibility studies. The phantom contains: 12.5 mM NAA, 10 mM creatine (Cr), 3 mM choline (Cho), 7.5 mM myo‐inositol (Ins), 12.5 mM glutamine (Glu), 5 mM lactate, 50 mM ${\text{KH}}_{2}{\text{PO}}_{4}$, 12.5 mM NaOH, 0.1% ${\text{NaN}}_{3}$, 1ml/L magnevist.

The spectra were acquired using similar acquisition parameters as for *in vivo* studies. The only difference refers to the number of signal averages: 64 for water suppressed spectra, and 8 for water unsuppressed ones. The spectra were acquired from the center of the phantom located in the scanner isocenter. The median time interval between the *in vitro* measurements was eight days (time interval range: 2‐90 days). The total number of short TE measurements was 118 and the total number of long TE measurements was 116. The room temperature was controlled before H1 MRS *in vitro* studies ($24^{{}^{\circ}}\pm 1.5^{{}^{\circ}}C$). The signal‐to‐noise ratios (SNRs) for metabolites were estimated by taking the ratio of the peak amplitude to the standard deviation of a metabolite free region of the spectrum.

The NAA *in vitro* metabolite levels output by LCModel (corrected for voxel size, TG, R1 and R2, and obtained using $f_{\text{calib}}=1$) were subjected to change‐point analysis to identify break points when the shifts in the phantom metabolite levels occurred.^(14)^ This method uses cumulative sum control charts and bootstrapping to detect changes in mean values (10000 bootstrap samples were applied in this work). Mean square error estimator was employed to estimate when the change has occurred. After detection of a change, the data are broken into two parts, one each side of the change‐point, and the analysis is repeated for each segment. The procedure continues until no break point is detected. The changes found on a first pass through the data are referred to as level 1 changes, the changes found on a second pass through the data are referred to as level 2 changes, and so forth. The level number indicates the importance of a change. Theoretically, only the data from the previous change and up to the next change should be used in detection of a break point. Changes detected early in the above procedure are most likely to be biased. Therefore, when the set of break points is found, they are reestimated using only surrounding data and changes identified as statistically insignificant are discarded.

On the basis of a change‐point analysis, the monitored time period was divided into several time intervals characterized by different coefficients $f_{T}$ computed as the ratios of the NAA level in the interval encompassing November 16, 2007 (determination of $f_{calb}$) to the NAA level in the specific time interval.

To rule out possible instability in NAA concentration in the phantom, a similar phantom containing 12.5 mM NAA dissolved in the same solvent as in the original one was prepared. H1 MRS spectrum (TE 35 ms, TR 1500 ms) was measured from this phantom just after its preparation, and the results were compared to those obtained with the phantom provided by the manufacturer. The NAA stability in the latter phantom was assumed to be evidenced by similarity of the levels of this metabolite in these two phantoms. These comparative measurements were performed on 24 October 2010.

Change‐point analysis was applied to the NAA *in vitro* levels because of the highest SNR of this metabolite. However, temporal variation of Cr and Cho was also evaluated and characterized by the coefficients of variations (CV, ratios of standard deviation to the mean). Additionally, variation coefficients of the water‐scaled NAA, Cr, and Cho levels are also presented.

## III. RESULTS

**Figure 1:**
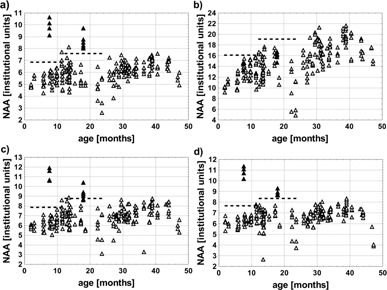
Water‐scaled NAA levels obtained from short TE H1 MRS *in vivo* spectra (a), water‐scaled NAA levels obtained from long TE H1 MRS *in vivo* spectra (b), NAA levels determined based on absolute standard (before application of $f_{T}$ coefficient) from short TE H1 MRS in vivo spectra (c), and NAA levels determined based on absolute standard (before application off $f_{T}$ coefficient) from long TE H1 MRS *in vivo* spectra (d). Full triangles represent two cases diagnosed with Canavan disease, open triangles represent patients diagnosed with other neurological disorders, dashed lines indicate the threshold of ‘mean + 2 SD’ computed separately for patients aged 2‐12 months and 12‐24 months. The NAA concentrations were not corrected for relaxation and are expressed in institutional units.

Figure 1 presents the NAA levels obtained using water scaling and absolute standard calibration (assuming $f_{T}=1$) in the studied neurological disorders. It is evident that the water‐scaled NAA levels determined from short TE spectra are increased in both cases of Canavan disease (z‐scores above 2 SD, Fig. 1(a)). However, the water‐scaled NAA levels determined from long TE spectra in this disorder fall within the range corresponding to other neurological disorders (z‐scores from 0.41 to 1.20 SD, Fig. 1(b)). Relaxation changes are expected to be responsible for these contradicting results. To eliminate the confounding effect of water relaxation changes the NAA levels determined based on the absolute standard calibration should be considered. Figures 1(c) and 1(d) show that the NAA z‐scores for case 1 are above 2 SD both in short (z‐scores from 5.8 to 7.6 SD) and long TE spectra (z‐scores from 6.0 to 7.9 SD). The difference between the NAA levels determined from two short TE spectra in case 2 and the NAA levels measured in other neurological disorders is slightly below the border value for statistical significance (z‐scores around 1.9 SD). The remaining short and long TE spectra reveal NAA z‐scores above 2 SD in this case (z‐scores from 2.1 to 3.5 SD in short TE spectra and from 2.3 to 3.0 SD in long TE spectra). However, reliable quantification of NAA levels based on the absolute standard method requires careful analysis of scanner performance reproducibility.

**Figure 2:**
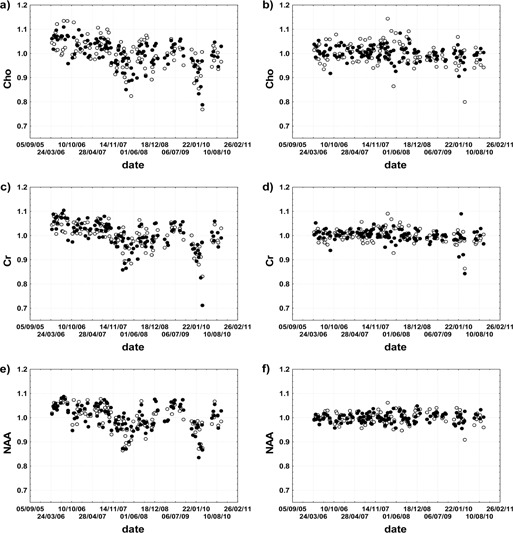
Temporal variability of metabolite levels obtained from H1 MRS *in vitro* measurements: Cho (a), Cr (c), and NAA (e) *in vitro* levels corrected for TG, R1, and R2; and Cho (b), Cr (d), and NAA (f) *in vitro* levels obtained using a water‐scaling technique. Dots represent short TE data, while circles represent long TE data. The metabolite levels are expressed relative to their mean levels in the period from April 2006 to September 2010.

Comparison of the in vitro reproducibility of metabolite levels corrected for TG, R1, and R2 with the results of water‐scaling technique is presented in Fig. 2. The coefficients of variation and the SNR parameters for NAA, Cho, and Cr are given in Table 1. The variation coefficients of metabolite concentrations corrected for TG, R1, and R2 vary from 5.41% to 5.96% for short TE spectra and from 4.84% to 6.19% for long TE spectra. The variation coefficients of metabolite concentrations obtained with the use of a water‐scaling technique fall within the range from 1.86% to 2.8% for short TE spectra and from 2.32% to 4.61% for long TE spectra.

**Table 1:** CVs and SNRs for the Cho, NAA, and Cr levels obtained from *in vitro* studies

*Metabolite*	*TE (ms)*	*SNR* (mean±standard deviation)	*CV of Metabolite Levels Corrected for TG, R1, R2 (%)*	*CV of Water Scaled Metabolite Levels (%)*
Cho	35	54±7	5.83	2.8
	144	33±6	6.19	4.61
NAA	35	144±20	5.41	1.86
	144	110±14	5.03	2.32
Cr	35	85±11	5.96	2.73
	144	62±11	4.84	2.73

**Figure 3:**
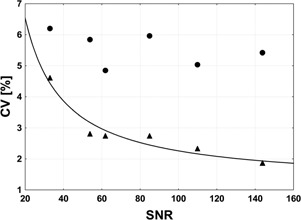
Relationship between CV and SNR for NAA, Cho, and Cr *in vitro* levels. Dots represent data corrected for TG, R1, and R2 and triangles represent data obtained using water‐scaling technique. Both short and long TE *in vitro* data are included (Table 1). Statistically significant relationship $\left(CV=\frac{106.9}{SNR}+1.2,p<0.01\right)$ was found between CV and SNR in water‐scaling technique.

Figure 3 shows the relationship between CV and SNR for the evaluated metabolites. B1 inhomogeneity, the variations in coil loading conditions, as well as in hardware performance, do not influence metabolite levels determined with the use of water‐scaling technique and a lower limit of reproducibility obtained in this method is set by SNR. We therefore expect CV of at least $\frac{100\%}{SNR}$. Of note, statistically significant relationship $\left(CV=\frac{106.9}{SNR}+1.2,p<0.01\right)$ was found between CV and SNR in water‐scaling technique. As expected, variability of metabolite levels corrected for TG, R1, and R2 is caused also by some different sources of error than noise in the spectra.

The measurements performed on October 24, 2010 confirmed the NAA stability in the phantom (the difference between the NAA levels obtained with two comparative measurements was 0.5%). Due to the highest SNR (Table 1), this metabolite was subjected to change‐point analysis.

Tables 2 and 3 present values of $f_{T}$ coefficients obtained from change‐point analysis of short and long TE data. Both sets of data indicate that the most important change occurred around October 30, 2007 ($f_{T}$ changed from 1.08 to 1.0). This change was detected on the first pass through the data and is described by a relatively narrow confidence interval. The change around July 23, 2008 detected in short TE data and the change around May 28, 2008 detected in long TE data are characterized by wide overlapping confidence intervals and cannot be accurately pinpointed. However, the magnitudes of these changes are lower than of that for October 30, 2007. The confidence intervals for three subsequent change‐points determined from short and long TE data also overlap, suggesting agreement between these two datasets. However, in the long TE data, a change around October 19, 2006 was detected which was not found in the short TE data. This change is, however, characterized by a wide confidence interval.

**Table 2:** The coefficients $f_{T}$ obtained from a change‐point analysis of *in vitro* NAA levels corrected for TG, R1, and R2 (short TE data) in the period from April 2006 to September 2010

*Change‐point*	*Confidence Interval*	*Confidence Level (%)*	*Change From*	*Change To*	*Level*
30 Oct 2007	10 Oct 2007 – 12 Dec 2007 (2.1 months)	100	1.08	1.0	1
23 Jul 2008	5 Dec 2007 – 26 Nov 2008 (11.9 months)	94	1.0	1.03	6
19 Dec 2008	20 Nov 2008 – 20 Apr 2009 (5 months)	100	1.03	1.09	7
10 Dec 2009	23 Sep 2009 – 22 Dec 2009 (3 months)	100	1.09	0.97	8
22 Jun 2010	17 Feb 2010 – 6 Jul 2010 (4.6 months)	95	0.97	1.06	6

**Table 3:** The coefficients *f*_*T*_ obtained from a change‐point analysis of *in vitro* NAA levels corrected for TG, R1, and R2 (long TE data) in the pe^T^riod from April 2006 to September 2010

*Change‐point*	*Confidence Interval*	*Confidence Level (%)*	*Change From*	*Change To*	*Level*
19 Oct 2006	27 Jul 2006 – 4 Apr 2007 (8.4 months)	98	1.11	1.08	6
30 Oct 2007	24 Oct 2007 – 19 Dec 2007 (1.9 months)	100	1.08	1.0	1
28 May 2008	12 Dec 2007 – 26 Nov 2008 (11.7 months)	96	1.0	1.03	5
10 Dec 2008	24 Sep 2008 – 17 Jan 2009 (3.8 months)	99	1.03	1.08	5
10 Dec 2009	23 Sep 2009 – 4 Jan 2010 (3.4 months)	100	1.08	1.0	5
6 Jul 2010	4 Jan 2010 – 18 Aug 2010 (7.5 months)	92	1.0	1.06	4

Summarizing, the monitored time period was divided into six time intervals based on short TE data (seven time intervals based on long TE data) characterized by $f_{T}$ coefficient ranging from 0.97 to 1.09 (based on short TE data) and from 1.0 to 1.11 (based on long TE data).

Tables 4 and 5 present the number of patients studied in the particular time periods determined from the change‐point analysis. As seen from these tables, the majority of the older group of patients (aged 12‐24 months) diagnosed with other neurological disorders were examined in the time periods characterized by higher scanner sensitivity than during the period covering case 2 examination. After application of $f_{T}$ correction factors determined from change‐point analysis of short TE *in vitro* data, all the NAA z‐scores for case 2 exceed the threshold of 2 SD (z‐scores from 2.27 to 4 SD for short TE data and from 2.9 to 3.6 SD for long TE data). The corrected z‐scores for case 1 fall within the range from 6.0 to 7.9 SD for short TE and from 6.0 to 8.0 SD for long TE data. Similar results are obtained when the correction factors determined from long TE *in vitro* data are applied.

**Table 4:** Distribution of examinations of patients diagnosed with Canavan disease and other neurological disorders among time periods determined from change‐point analysis of short TE *in vitro* data

		*Examinations of Patients Diagnosed with*:			
		*Canavan Disease*		*Other Neurological Disorders*	
*Time Period*	$f_{T}$	*Case 1*	*Case 2*	*Aged 2‐12 months*	*Aged 12‐24 months*
3 Apr 06 – 30 Oct 07	1.08	+		6	6
31 Oct 07 – 23 Jul 08	1.0		+	5	2
24 Jul 08 – 19 Dec 08	1.03			1	‐
20 Dec 08 – 10 Dec 09	1.09			2	1

**Table 5:** Distribution of examinations of patients diagnosed with Canavan disease and other neurological disorders among time periods determined from change‐point analysis of long TE *in vitro* data

		*Examinations of Patients Diagnosed with*:			
		*Canavan Disease*		*Other Neurological Disorders*	
*Time Period*	$f_{T}$	*Case 1*	*Case 2*	*Aged 2‐12 months*	*Aged 12‐24 months*
3 Apr 06 – 19 Oct 06	1.11			1	‐
20 Oct 06 – 30 Oct 07	1.08	+		5	6
31 Oct 07 – 28 May 08	1.0		+	3	2
29 May 08 – 10 Dec 08	1.03			3	‐
11 Dec 08 – 10 Dec 09	1.08			2	1

## IV. DISCUSSION

Canavan disease is a rare form of leukodystrophy caused by a deficiency of aspartoacylase — an enzyme hydrolyzing NAA to acetate and aspartate. This metabolic defect leads to accumulation of NAA in plasma, urine, and brain. As NAA is the most prominent signal in H1 MRS *in vivo* spectra of the normal brain, this method is believed to provide useful diagnostic information in this condition. The high NAA‐to‐Cr or NAA‐to‐Cho ratios are widely documented metabolic spectroscopic findings in this disease.^22^, ^23^ However, the results of absolute NAA quantification should be interpreted with caution. The concentration of NAA measured using H1 MRS with respect to the wet weight of brain tissue or to the brain volume is expected to be underestimated due to reduced cellularity and increased water content observed in this disease. Both normal^(23)^ and increased^(22)^ NAA concentrations were reported in Canavan disease using fully relaxed water signal as an internal standard. In our work, interpretation of water‐scaled metabolite levels is even more difficult due to lack of relaxation corrections. Of note, these corrections are rarely performed in a routine clinical practice. Since long TE spectra are more vulnerable to changes in T2 relaxation times than short TE ones, we observed that the NAA level obtained from long TE spectra in Canavan disease falls within the range corresponding to other neurological disorders. The NAA level obtained by means of absolute reference technique was found to be increased both in short and long TE spectra. However, reliability of metabolite concentrations obtained using this technique depends on the location‐dependent coil B1 variability, adequate loading correction, and adjustment for temporal changes in the scanner performance.

According to LCModel manual, automatically determined TG can be used for computation of absolute metabolite concentrations at GE 1.5 T scanner.^(18)^ This parameter is widely used for coil load correction,^24^, ^25^ but it does not account for local B1 inhomogeneities. The location dependent B1 variability is especially problematic in the analysis of interregional metabolic differences. In this study, the spectra acquired from several white matter localizations were pooled in the analysis to assess global — rather than location‐specific — metabolic changes in CD, so incorporation of B1 inhomogeneity correction was not a critical step in our work. Similarly, Hájek et al.,^(12)^ Schirmer and Auer,^(10)^ and Brooks et al.^(5)^ did not consider B1 inhomogeneity corrections in their studies devoted to evaluation of reproducibility of absolute reference technique at 1.5 T. However, fitting of the localized signal to the flip angle variation around 90° to account both for loading effects and B1 inhomogeneities would increase the precision of absolute reference technique and should be considered as a valuable modification of the methods presented in our work.^26^, ^27^

The most critical point in our work was to use the *in vitro* measurements to ensure that the *in vivo* NAA increase observed in two cases of Canavan disease represents true pathology and is not hardware related. Similarly, Soreni et al.^(13)^ used phantom measurements to rule out the risk that systematic variation due to scanner performance accounts for the changes observed in repeated measurements of striatal NAA levels. Long‐term monitoring of scanner stability over a one‐year period made it possible to increase the precision of *in vivo* metabolite levels normalized based upon the principle of reciprocity by about 4% in the study of Hajek et al.^(12)^ Breakdown of MR system and replacement of the coil and preamplifier were reported to be associated with the most significant changes in $f_{T}$ in the Hajek study (up to around 50%). In our work, long‐term variation was considerably lower. Despite longer time of scanner evaluation, neither head coil nor the preamplifier was replaced during this period. We could tentatively correlate the change‐point around October 19, 2006 (seen only in long TE data) with the only breakdown of the system in the monitored period, and the change‐point around October 10, 2008 with the system upgrade. It is also possible that some change‐points (for example around October 30, 2007) were influenced to some degree by service engineers' interventions. However, precise correlation of the changes observed in our *in vitro* spectroscopic measurements with the actual changes in hardware performance would require more elaborated and time‐consuming quality control procedures performed in parallel with the presented ones.^(28)^ Although service engineers usually check the performance of MRI systems during the routine visits, Sobol et al.^(29)^ found that 80% of the independently evaluated systems revealed deficiencies affecting image quality. Moreover, engineers are mainly focused on obtaining agreement with specified parameters, while the user may wish to observe any drifts in the scanner performance affecting absolute quantification. Although there were not many ‘natural’ break points in our study (no hardware parts replacement and only one breakdown of the system), change‐point analysis was successful in detection of subtle changes. Time‐dependent shifts in the *in vitro* NAA levels are sensitive, although not specific, indicators of changes affecting directly absolute metabolite quantification such as resonance frequency, flip angles, coil tuning, loading correction, gradient calibration, and receiver gain settings. Since all of these factors are important in metabolite quantification, periodical monitoring of scanner reproducibility presented in our work should facilitate this task and is well‐suited for a busy clinical scanner. Although traditional control charts are preferred for online assessment of metabolite levels change‐point analysis is better suited for analysis of historical data.^(14)^ Thus, application of this technique for retrospective analysis of large datasets collected during several years is fully justified.

Although phantom measurements cannot reproduce conditions encountered in *in vivo* studies, they constitute a valuable source of information about the best possible reproducibility. Schirmer and Auer^(10)^ measured short TE spectra from a brain phantom twice a week over a period of 13 months. Their coefficients of variation for the NAA, Cho, and Cr concentrations obtained based on the principle of reciprocity were 3.3%, 3.8%, and 4%, respectively. Simmons et al.^(11)^ evaluated variability in metabolite concentrations obtained from long TE spectra scaled to water over a period of two years and found that the coefficients of variation for NAA, Cho, and Cr were 1.5%, 2.4%, and 1.5%, respectively. Comparing these findings with ours, it should be emphasized that the results of reproducibility measurements are highly scanner‐specific and depend also on the acquisition parameters influencing SNR (for example, both Schirmer and Simmons studies used higher number of spectra averages; furthermore, the Simmons study applied longer TR).

## V. CONCLUSIONS

This work presents the usefulness of long‐term *in vitro* MR system reproducibility monitoring in interpretation of *in vivo* results in rare white matter disorders. This is the first report of combining unsupervised change‐point detection technique with spectroscopic phantom measurements in quality control of an MRS system. Taking technical complexity of MRS systems into account, application of powerful unsupervised techniques facilitates absolute quantification of metabolites.
